# Stigma Among the Informed–Lived Experience of South Asians Navigating Stigma From the Mentally Literate

**DOI:** 10.1192/bjo.2026.11192

**Published:** 2026-06-30

**Authors:** Aishath Shahama, Aminath Nausha Ahmed, Jerome Carson

**Affiliations:** 1 University of Greater Manchester, Bolton, Greater Manchester, United Kingdom; 2 Somerset NHS Foundation Trust, Taunton, United Kingdom

## Abstract

**Aims:**

Literature identifies South Asians as reporting significantly higher rates of mental health stigma compared to other ethnic groups. A significant component of this is ‘courtesy stigma’, where family members experience collective shame regarding an individual’s diagnosis. While low Mental Health Literacy (MHL) is cited as a primary driver of stigma, this study investigates the paradox: the persistence of stigma among ‘mentally literate’ South Asian households. The study aims to explore the lived experience of individuals navigating mental health while being raised by parents with high MHL, questioning whether MHL effectively translates into appropriate support.

**Methods:**

This qualitative study utilizes semi-structured interviews via Zoom (Howe, Tickle & Brown, 2014). Participants were recruited through snowball sampling for their unique perspectives: All participants (2 males, 4 females) have parents with professional healthcare backgrounds or are in the field of psychology. Data was analysed using narrative thematic analysis to capture the various stigmas faced within familial dynamics.

**Results:**

Four core themes emerged: Denial, Courtesy Stigma and Labelling, Paradoxical Resistance to Treatment, and Stigmatised Protection and Exclusion. Denial was most prominent, where a psychiatrist father’s clinical expertise failed to overcome a “refusal to see” of his son’s diagnosis. Courtesy Stigma and Labelling, for example where the “mad person’s daughter” label, created such a traumatic environment of social shaming. Paradoxical Resistance to Treatment, where even high levels of MHL fail to ensure support. A pharmacist,well-versed in the efficacy of medication, discouraged treatment due to the specific stigma of psychiatric drugs. Noteworthy to mention an exceptional instance of supportive parents, including a psychiatrist father who encouraged medical treatment for her OCD from a young age, the participant still felt stigmatised. Stigmatised Protection and Exclusion, were evident when adults discouraged younger family members from interacting with the participants, ultimately leading to social exclusion.

**Conclusion:**

This case series demonstrates that stigma within South Asian communities remains a structural force dictating family behaviour independent of clinical knowledge. MHL does not inherently mitigate cultural shame. Clinicians, for instance, should not assume that parents with medical knowledge will be more accepting of a child’s diagnosis. Recognising this will be key in tailoring effective, culturally sensitive care. Larger-scale studies are required to fully understand the broader population’s cultural barriers and the persistence of stigma in “literate” households. Several participants have channelled their experiences with stigma into advocacy, pursuing careers in psychology or engaging in public awareness efforts to bridge these gaps.

